# Enhancement in Site-Specific Delivery of Carvacrol against Methicillin Resistant *Staphylococcus aureus* Induced Skin Infections Using Enzyme Responsive Nanoparticles: A Proof of Concept Study

**DOI:** 10.3390/pharmaceutics11110606

**Published:** 2019-11-13

**Authors:** Maria Mir, Naveed Ahmed, Andi Dian Permana, Aoife Maria Rodgers, Ryan F. Donnelly, Asim.ur. Rehman

**Affiliations:** 1 Department of Pharmacy, Faculty of Biological Sciences, Quaid-i-Azam University, Islamabad 45320, Pakistan; mariamir@bs.qau.edu.pk (M.M.); natanoli@qau.edu.pk (N.A.); 2 School of Pharmacy, Medical Biology Centre, Queen’s University Belfast, 97 Lisburn Road, Belfast BT9 7BL, UK; apermana01@qub.ac.uk (A.D.P.); arodgers11@qub.ac.uk (A.M.R.); 3 Department of Pharmaceutics, Faculty of Pharmacy, Hasanuddin University, Makassar 90234, Indonesia

**Keywords:** carvacrol, poly(ε-caprolactone), nanoparticles, methicillin resistant *Staphylococcus aureus* (MRSA), enzyme responsive system, stimuli responsive drug delivery

## Abstract

Methicillin resistant *Staphylococcus aureus* (MRSA) induced skin infections have become a challenging problem due to the escalating antibiotic resistance. Carvacrol (CAR) has been reported to be effective against MRSA. However, due to its characteristics, CAR exhibits low skin retention. In this study, CAR was formulated into site-specific nanoparticle (NPs) delivery system using poly(ε-caprolactone) (PCL), following incorporation into a hydrogel matrix to facilitate dermal delivery. The release study exhibited significantly higher release of CAR from PCL NPs in the presence of bacterial lipase, highlighting its potential for differential delivery. Moreover, encapsulation of CAR in PCL NPs resulted in a two-fold increase in its anti-MRSA activity. Dermatokinetic studies revealed that the NPs loaded hydrogel was able to enhance skin retention of CAR after 24 h (83.29 ± 3.15%), compared to free CAR-loaded hydrogel (0.85 ± 0.14%). Importantly, this novel approach exhibited effective antimicrobial activity in an ex-vivo skin infection model. Hence, these findings have proven the concept that the loading of CAR into a responsive NPs system can lead to sustained antimicrobial effect at the desired site, and may provide a novel effective approach for treatment of MRSA induced skin infections. However, further studies must be conducted to investigate in-vivo efficacy of the developed system in an appropriate infection model.

## 1. Introduction

*Staphylococcus aureus* (*S. aureus*) is both a commensal bacterium and a human pathogen. Multidrug-resistant *S. aureus*, specifically methicillin-resistant *S. aureus* (MRSA), remains one of the dominant causes of infection related mortality [1]. It is the main cause of nosocomial infections, but the frequency of occurrence of community-acquired infections is also increasing [2]. MRSA colonization is a major risk factor for adverse health outcomes, with approximately 10–30% of carriers subsequently developing infection, both immediately after colonization, or in the year following the identification of carriage status [3]. MRSA causes a plethora of difficult to treat infections, including pneumonia, osteomyelitis, endocarditis, emphysema, toxic shock syndrome, and skin and soft tissue infections (SSTIs) [4]. Among these infections, SSTIs have been rising dramatically since the last 10 years, presenting higher rates of complications. MRSA is found to be the principal causative agent in approximately 90% of SSTIs [5,6]. It has emerged resistance to commonly used antibiotics, including the aminoglycosides, macrolides, fluoroquinolones, chloramphenicols, and tetracyclines [7]. In recent years, quinupristin, linezolid, daptomycin, and tigecycline have been approved for treatment of MRSA infections. More recently, however, studies have reported the emergence of resistance to linezolid, vancomycin, and daptomycin [8,9]. Moreover, resistance to mupirocin, the most successful topical antibiotic for clearance of nasal MRSA, reportedly ranges from 1–81% [3]. With limited treatment options available to manage MRSA induced infections and the concomitant rise in multidrug resistance, there is a requirement for identification of alternative agents for management of these often life-threatening infections [10].

Essential oils and their bioactive molecules have been reported to exhibit antimicrobial properties against a range of Gram-positive and Gram-negative pathogenic bacteria [11,12]. Among these, carvacrol (CAR), 5-isopropyl-2-methylphenol, a monoterpenoid phenolic compound, and the principal component of many plant essential oils, has manifested antimicrobial potential against a broad range of micro-organisms, including MRSA [13]. It is present in the essential oils of *Citrus bergamia*, *Origanum vulgare*, *Trachyspermum ammi*, *Thymus vulgaris*, and *Lepidium africanum*. CAR can penetrate bacterial cell membranes, resulting in loss of membrane integrity and subsequent release of bacterial cell components [12,14]. Specifically, it causes disruption of proton gradient, subsequently upsetting adenosine triphosphate (ATP) synthesis, inhibits efflux pumps, produces reactive oxygen species, and suppresses two essential virulence factors, namely enterotoxin and coagulase. It can also inhibit quorum sensing by impeding the autoinducer-2 (AI-2) system and inhibiting *S. aureus* biofilm formation [15,16]. In-spite of the promising antimicrobial profile of CAR, its direct application still encounters challenges owing to its permeability, that leads to poor skin retention, requiring frequent applications. Therefore, development of an optimized delivery system is required to achieve sustained antimicrobial effect at the site of infection [17,18].

Biodegradable polymeric nanoparticles (NPs) are being used as a carrier drug delivery system for a variety of bioactive molecules, due to their controlled release properties, biocompatibility, stability, and non-immunogenic nature [19]. Moreover, these NPs delivery systems can be tailored to respond to the unique microenvironment of diseased sites, enabling on-demand drug delivery at the site of infection [20]. For example, infected tissues have higher expression of specific enzymes, lower pH, secreted toxins, bacterial antigens, and surface charges, all of which can be exploited using NPs for differential drug delivery [21]. Xiong et al. has recently reported the chemical synthesis of bacterial lipase-sensitive poly(ε-caprolactone) (PCL) nanogel for specific delivery of antimicrobials to infection sites. Importantly, study results demonstrated that delivery system released the drug, only in the presence of lipase producing *S.aureus* strains [22]. Thus, production of lipase at infection site may be utilized to selectively deliver antimicrobials for the management of infections by lipase producing bacterial strains and may prove an effective, safe treatment approach.

The selection of final dosage form, in terms of its suitability for application and patient acceptability, is as important as designing of delivery system. Considering the potential of hydrogel to stabilize and protect the wound, maintaining a balance in moisture content (where excess exudates are absorbed in wet wounds and water is provided to dry wounds), and gentle to skin upon application, hydrogel was considered to be the optimum choice for delivery of designed NPs to infected skin [23]. Among variety of polymers, carbopol was selected owing to its several attractive features including; well-known and well-characterized pharmaceutical ingredient in many skin products, lower risk of having contaminants in comparison with the polymers of natural origin, low toxicity and ability to form pharmaceutically elegant gels [24].

Herein, we report for the first time the design, optimization and characterization of NPs laden with CAR for potential treatment of MRSA induced skin infections. NPs were fabricated from the bacterial lipase-sensitive poly(ε-caprolactone) polymer. It is anticipated that encapsulation of CAR within NPs, would improve CAR stability and prevent nonspecific release, while providing the opportunity to selectively deliver NPs to infected tissue through NPs degradation by bacterial lipases at infection sites. For effective delivery of designed NPs to infected skin, these were further incorporated into hydrogel. In order to investigate the concept that loading of CAR into PCL NPs can prolong the availability of CAR in skin layers, in addition to improving its antibacterial activity, the following studies were conducted; ex-vivo dermatokinetic profiling, skin deposition and distribution studies, and antimicrobial efficacy studies in a skin wound model. This is the first reported study utilizing this approach for enhancement of delivery of CAR.

## 2. Materials and Methods

### 2.1. Materials

Poly(ε-caprolactone) (PCL) (MW = 14,000 Da), carvacrol (>98% purity), Poloxamer^®^ 407, Poly(vinyl alcohol)(PVA) (MW = 36,000 Da), Carbopol^®^ 934, acetone (>99.5%), Mueller Hinton Broth and lipase from *Pseudomonas cepacia* (≥30 U/mg) were procured from Sigma Aldrich, Hamburg, Germany. Dimethyl sulfoxide (DMSO) and triethanolamine were obtained from Merck kGaA, Darmstadt, Germany. Gluteraldehyde was obtained from Daejung Chemicals and Metals Co., Ltd. Shiheung, Korea. All reagents used were of analytical grade.

### 2.2. Preparation of Blank and CAR-PCL NPs

**Figure 1:**
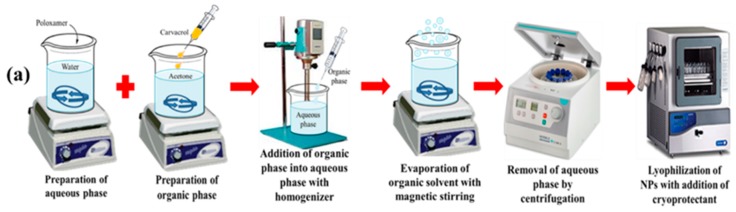

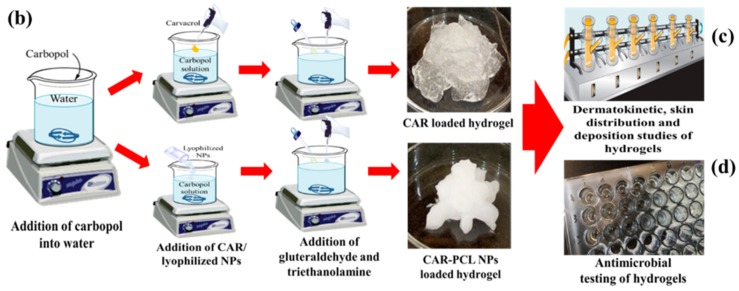
(**a**) Schematic illustration of preparation of CAR-PCL NPs; (**b**) preparation of CAR and CAR-PCL NPs loaded hydrogel; (**c**) ex-vivo dermatokinetic, skin distribution, and deposition studies; and (**d**) indication of antimicrobial testing of CAR and CAR-PCL NPs loaded hydrogel. CAR, carvacrol; PCL, poly(ε-caprolactone); NPs, nanoparticles.

Blank and CAR-PCL NPs were prepared using nanoprecipitation, as previously described [25]. Briefly, PCL was dissolved in 4 mL of acetone in predetermined concentrations with mild heating. This polymer solution was added to 12 mL of milli-Q water at a rate of 0.16 mL/minute containing hydrophilic surfactant under homogenization at 6500 rpm (Silent Crusher M Homogenizer from Heidolph Instruments, Schwabach, Germany). Three different surfactants, namely Tween^®^ 80, PVA and Poloxamer^®^ 407, were tested in a concentration range of 0.5% to 2.5% for optimization of CAR-PCL NPs formulation. Organic solvent was evaporated at ambient temperature with continuous stirring for 40 min at magnetic stirrer (MSHP 1A 111, PCSIR, Islamabad, Pakistan). The resultant formulation was collected by centrifugation (Hermle Labortechnik GmbH, Z-206A, Siemensstr.25 D-78564 Wehingen, Germany) at 13,500 rpm (22413 RCF) for 30 min at 4 °C. Supernatant was removed and the resultant pellet was washed twice with milli-Q water and centrifuged for 30 min, as previously described. The pellet was frozen at −80 °C, before lyophilization (Alpha 1-2 LD plus, Christ, Osterode, Germany). NPs laden with CAR were formulated as above, with the addition of CAR in predetermined concentrations to the organic phase during formulation. Figure 1a presents the various steps involved in the formulation of NPs.

### 2.3. Optimization of CAR-PCL NPs through Experimental Design

Design Expert^®^ (version 7, Stat-Ease, Inc. 2021 East Hennepin Ave., Suite 480 software) was used to develop CAR-PCL NPs by optimizing the influential parameters of nanoprecipitation method. Central Composite Design (CCD), being considered as most appropriate for 3 factors under response surface methodology (RSM) was employed. Concentration of PCL (*X*_1_), surfactant (*X*_2_) (Poloxamer^®^ 407 was selected among other surfactants for further use on the basis of results of pre-formulation screening, data not shown) and CAR (*X*_3_) were taken as independent variables, while keeping the ratio of organic to aqueous phase constant i.e., 1:3. Eleven runs were executed by the software for given values (also determined experimentally in pre-formulation screening, data not shown) of low and high levels of these factors represented by −1 and +1, respectively. Nanoparticle size (*Y*_1_) and entrapment efficiency (EE %) (*Y*_2_) were taken as dependent variables for optimization of CAR-PCL NPs. Particulars of dependent and independent variables are given in Table S1. Each run was performed and values of dependent variables were determined for each trial. The composition of eleven formulations with obtained values of dependent responses is presented in Table 1.

### 2.4. Physicochemical Characterization of CAR-PCL NPs

#### 2.4.1. Particle Size, Morphology, Polydispersity Index and Zeta Potential

Both blank and CAR-PCL NPs were characterized in terms of particle size, polydispersity index (PDI), and zeta potential (ZP) by using zetasizer (Malvern, Nano ZS-90, Worcestershire, UK) at 25 ± 2 °C. The shape of CAR-PCL NPs was analyzed using transmission electron microscopy (TEM) (Joel JEM 1400 Plus Transmission Electron Microscope, Peabody, MA, USA). For measurement purpose, nanoparticles were stained with phosphotungstic acid.

#### 2.4.2. Estimation of Entrapment Efficiency and Percentage Yield

The entrapment efficiency (EE) of CAR-PCL NPs was estimated using a method as previously described [26]. CAR-PCL NPs formulations were centrifuged at 13,500 rpm (22413 RCF), at 4 °C for 30 min to separate the CAR-PCL NPs from the free drug. After collection, filtered supernatant (through 0.45 µm syringe filter) was assessed using a UV-Visible spectrophotometer (Dynamica, Halo DB-20, Newport Pagnell, UK) at 275 nm. Absorbance correction was conducted for the background absorbance of PCL. The following equation was used to determine EE:

(1) $$\mathrm{Entrapment}\;\mathrm{efficiency}\;\left(\mathrm{EE}\right)\%=\frac{\mathrm{Total}\;\mathrm{CAR}\;\mathrm{added}-\mathrm{Free}\;\mathrm{CAR}\;\mathrm{in}\;\mathrm{supernatant}}{\mathrm{Total}\;\mathrm{CAR}\;\mathrm{added}}\times 100$$

The total concentration of lyophilized nanoparticles in relation to initial concentrations of drug and polymer used was measured to determine the percentage yield of CAR-PCL NPs. The following formula was used to calculate percentage yield [27]:

(2) $$\%\;\mathrm{Yield}\;=\;\frac{\mathrm{Mass}\;\mathrm{of}\;\mathrm{dried}\;\mathrm{nanoparticles}}{\mathrm{Total}\;\mathrm{mass}\;\mathrm{of}\;\mathrm{CAR}+\mathrm{PCL}\;}\times 100$$

### 2.5. Fourier Transform-Infrared (FTIR) Spectroscopy

Compatibility study of ingredients used in development of CAR-PCL NPs was performed through FTIR analysis [28]. CAR, PCL, Poloxamer^®^ and CAR-PCL NPs were ingrained with KBr disc prior to evaluation through FTIR spectrophotometer (100 L160000A, Perkin Elmer, Waltham, MA, USA).

### 2.6. In-Vitro Release Kinetic Study of CAR-PCL NPs

In-vitro release behavior of CAR from CAR-PCL NPs and in pure form was determined using the dialysis bag diffusion method [29]. To evaluate the bacterial enzyme responsive release of the developed CAR-PCL NPs, release study was conducted at different pH conditions, in both the absence and presence of *Pseudomonas lipase* [22]. For this purpose, free CAR and NPs were dispersed in 1 mL of phosphate buffer and added to a dialysis membrane tubing (molecular weight cutoff 12–14 kDa), in the presence or absence of bacterial lipase (0.5 or 1 mg/mL). This was then immersed in phosphate buffer (100 mL) at different pH to represent different conditions, specifically, phosphate buffer pH 5.5 (normal skin), 6.5 (infection site/wounds), and 7.4 (physiological medium). The release study was run in a shaking water bath (Memmert, water bath, WNB 14, Schwabach, Germany), set at 30 cycles/minute frequency and 37 ± 1 °C temperature. Samples (1 mL volumes) were taken at pre-determined time points (0.5, 1, 2, 4, 6, 8, 12, 24, and 48 h) and replaced with fresh phosphate buffer. Subsequent analysis of collected samples using UV-Vis spectrophotometer was performed at 275 nm. Results were obtained in triplicate (*n* = 3) and expressed as mean ± standard deviation (SD). Cumulative release % was determined through following formula:

(3) $$\mathrm{Cumulative}\;\mathrm{release}\;\left(\%\right)=Ct.Vo+\overset{t-1}{\underset{i=0}{\sum }}Vi\times 100/\mathrm{CAR}\;\left(\mathrm{initial}\;\mathrm{conc}\right)$$

Where: *V*o = initial buffer volume, *V*i = volume of sample taken, and *C*t = CAR concentration in each sample obtained through UV.

The mechanism of release of CAR from CAR-PCL NPs was studied by applying different mathematical models including; zero-order, first-order, Hixson–Crowell, and Higuchi and Korsmeyer–Peppas on in-vitro data using DD Solver^®^ software. Significant difference between multiple groups was determined through one-way analysis of variance (ANOVA) followed by Bonferroni’s multiple comparison test using GraphPad Prism^®^ software version 5.03 (GraphPad Software, San Diego, CA, USA).

### 2.7. In-Vitro Antibacterial Assays

#### 2.7.1. Culture of MRSA Strains

American Type Culture Collection (ATCC) strains used in current study were directly purchased from LGC Standards UK. Both MRSA strains; ATCC^®^ 33593^TM^ and ATCC^®^ BAA-1707^TM^ (MW2) were maintained at 4 °C and sub-cultured at regular intervals on fresh media. Before each antimicrobial assay, bacteria were cultured overnight in Mueller Hinton broth (MHB) at 37 °C, 100 rpm, and then the resulting culture was centrifuged at 4000 rpm for 20 min to get a pellet. The obtained pellet was resuspended in fresh MHB and the optical density OD_550_ of bacterial suspension was set to 0.2 equivalents to 1.5 × 10^8^ CFU/mL (precise bacterial count was determined by plating the diluted suspension on Mueller Hinton agar (MHA) plates). Working inoculums were established by multistep dilutions with MHB to obtain final concentrations of 2.0 × 10^5^ CFU/mL.

#### 2.7.2. Evaluation of Lipase Producing Activity

In order to confirm the lipase producing activity of MRSA strains, a precipitation test utilizing Tween^®^ 80 substrate plates was performed. The recipe used for preparation of Tween^®^ 80 agar plates was (*w/v* %); 1.5% peptone, 0.1% calcium chloride, 0.5% sodium chloride, 1% Tween^®^ 80, and 1.5% agar. Bacterial suspension was prepared and adjusted to the required concentration following the same method mentioned in Section 2.7.1, and inoculated into the plates followed by incubation at 37 °C for 24 h. The white precipitation surrounding the inoculated colonies was confirmation of lipase activity.

#### 2.7.3. Determination of Minimum Inhibitory Concentration and Minimum Bactericidal Concentration

Minimum inhibitory concentrations (MICs) of CAR, blank PCL NPs and CAR-PCL NPs were determined against MRSA strains; ATCC^®^ 33593^TM^ and ATCC^®^ BAA-1707^TM^ (MW2) through the microtiter broth dilution method using 96-well plates as previously described [30]. Two-fold serial dilutions of CAR, blank PCL NPs and CAR-PCL NPs were made with MHB to achieve final concentrations ranging from 2.5 mg/mL to 0.039 mg/mL. 100 µL of each bacterial suspension was inoculated to all wells to obtain final bacterial concentration of 2.0 × 10^5^ CFU/mL. MHB with inoculum without any antibacterial agent was included as positive control. The inoculated microplates were incubated at 37 °C for 24 h. MIC was estimated as the minimum concentration of CAR, blank PCL NPs and CAR-PCL NPs completely inhibiting the visible growth of bacteria following overnight incubation.

To estimate minimum bactericidal concentration (MBC), 20µL from each well exhibiting no visible growth was cultured on MHA plates and incubated at 37 °C for 24 h. The minimum concentration of CAR and CAR-PCL NPs causing >99.9% inhibition of bacterial growth was considered as the MBC. Three inter-day and intraday replications were performed for both MICs and MBCs determinations.

#### 2.7.4. Killing Kinetics of CAR and CAR-PCL NPs

Killing kinetics of CAR and CAR-PCL NPs against MRSA strains were estimated following the method previously reported [31]. Briefly, the concentration of MRSA test strains was set to 2.0 × 10^5^ CFU/mL following the same way mentioned in Section 2.7.1. Bacterial suspensions were then treated with CAR and CAR-PCL NPs in concentrations equivalent to their MIC, 2 × MIC, 4 × MIC and 8 × MIC followed by incubation at 37 °C. Samples from the cultures were withdrawn at 0, 4, 6, 8, 12, 18, and 24 h incubation time points and inoculated on MHA plates with appropriate dilutions. Plates were analyzed for viable CFUs after 24 h of incubation at 37 °C. A control was also run under the same conditions (without CAR and CAR-PCL NPs) for MRSA growth at all time points. The CFUs retrieved post-treatment were compared for both CAR and CAR-PCL NPs and plotted as the log CFU/mL against post-treatment time. The relationship between viable cell count and time was evaluated through a time-kill curve. Experiments were performed in three replications.

### 2.8. Preparation of CAR and CAR-PCL NPs Loaded Hydrogel

To facilitate topical drug delivery, the optimized CAR-PCL NPs were loaded into a 1% *w/v* Carpobol^®^ 934 hydrogel to obtain a rheologically suitable formulation. Carbopol was dissolved in distilled water to avoid agglomeration; with continuous stirring at 300–350 rpm for 1–2 h. Lyophilized CAR-PCL NPs (15.2% *w/v*) were added in carbopol solution and mixed completely. After complete homogenization of CAR-PCL NPs, 0.03% *v/v* (of whole formulation) glutaraldehyde was added as a cross-linker. Finally, triethanolamine was dropwise added to achieve neutral pH. Similarly, CAR loaded hydrogel was also prepared as a control, following the aforementioned protocol with addition of CAR (0.7% *w/v*) in place of CAR-PCL NPs. A schematic illustration of steps involved in the formulation of hydrogels is given in Figure 1b.

### 2.9. Characterization of CAR-PCL NPs Loaded Hydrogel

#### 2.9.1. Physical Appearance, pH, Drug Content and Spreadability of Hydrogel

Hydrogels were visually inspected for their appearance, clarity, distribution, and non- grittiness. pH of hydrogel was determined by dipping probe of pH meter (PHS25CW Bante instrument Chicago, IL, USA) directly in the formulation. For estimation of CAR concentration in hydrogel, 0.5 g of final formulation was dissolved in 5 mL of phosphate buffer with sonication at 37 °C for 30 min. The resultant mix was subsequently filtered using 0.45 µm syringe filter and analyzed at 275 nm wavelength on UV-Vis spectrophotometer, after making suitable dilutions with phosphate buffer (pH 7.4). Formula used to calculate % drug content is given below:

(4) $$\mathrm{CAR}\;\mathrm{content}\;\left(\%\right)=\;\frac{\mathrm{Observed}\;\mathrm{quantity}\;\mathrm{of}\;\mathrm{CAR}}{\mathrm{Theoretical}\;\mathrm{quantity}\;\mathrm{of}\;\mathrm{CAR}\;\mathrm{in}\;\mathrm{hydrogel}}$$

To determine spreadability of the hydrogel the glass slide method was employed [32]. Briefly, two glass slides were used and center of one slide was marked with a circle of 1 cm diameter. Gel (0.5 g) was added to the circular area and allowed to spread. When there was no further spreading of the gel, the second glass slide was put on the first one, in a way that the gel became sandwiched between two slides. A mass of 500 g was placed on the upper slide and the resulting increase in diameter of the gel was recorded.

#### 2.9.2. Rheological Evaluation

The rheological studies were performed by utilizing cone plate rheometer (DV3T-Brookfield Rheometer, AMETEK, Brookfield, MA, USA) at 25 ± 2 °C. A small amount of gel was placed on a plate and measurements were taken at varying shear speeds (0.5–12 rpm). Viscosity (cP) of hydrogel at various speeds was measured to establish the flow pattern of the formulation [33].

#### 2.9.3. Extrudability Measurement

The extrudability test was performed by employing a hardness tester (TBH 125, ERWEKA GmbH, Langen, Germany). A collapsible aluminum tube open from the back end was taken and 15 g of hydrogel was filled in it. After filling the tube, the back end was properly crimped to avoid roll back of gel. The tube was properly adjusted in a tester and 1 kg/cm^2^ pressure was applied for 30 s to extrude the gel. The amount of the extruded gel was weighed after collection and the percentage of extruded gel was calculated (>90% = excellent extrudability, >80% = good extrudability, and >70% = fair extrudability) [34].

#### 2.9.4. Determination of Bioadhesion Time

In order to determine the time each formulation could remain adherent to the skin, disintegration test equipment (model PTZ-S, Pharma test, Siemensstr.5, Hainburg, Germany) was utilized. Phosphate buffer (pH 5.5, maintained at 32 °C) was filled in a beaker of disintegration apparatus. Pig skin was attached to the glass slide with help of thread and then this slide was carefully attached to a basket rack assembly using thread and adhesive tape. Hydrogel was applied on the skin and then the basket rack assembly was allowed to move up and down after immersing in phosphate buffer (pH 5.5). The time for the complete removal of gel was recorded [35].

### 2.10. Stability Studies of Hydrogel

CAR-PCL NPs loaded hydrogel was filled in scintillation glass vials and incubated at 25 °C (room temperature), 2–8 °C (refrigeration temperature), and at 40 ± 2 °C with 75 ± 5% relative humidity (accelerated conditions) for three months. Formulations were evaluated for differences in physical appearance (color change, synerisis/phase separation, and grittiness), particle size, pH, and drug content. For the purpose, samples were collected at pre-specified times and evaluated for essential parameters.

### 2.11. Ex-Vivo Dermatokinetic, Skin Deposition, and Distribution Studies

#### 2.11.1. Preparation of Skin Samples

For all ex-vivo studies, full-thickness pig skin was used. For the purpose, skin was excised from abdominal part of still born piglets within 24 h of post-mortem. Subcutaneous fat was separated using a scalpel and skin was then stored in petri plates at −20 °C till further use. Before starting experiments, skin was thawed in PBS (pH 7.4) for 30 min and hair was removed with a disposable razor.

#### 2.11.2. Dermatokinetic and Permeation Studies

Dermatokinetic and permeation studies were conducted on full thickness abdominal pig skin using Franz cells having an area of 1.76 cm^2^ for diffusion following the previously reported method [36]. Phosphate buffer (pH 7.4) was added in the receiving compartment and maintained at 37 ± 1 °C temperature with 600 rpm stirring. Pig skin was placed in the donor compartment and hydrogels were applied on the skin. Both the sampling arm and donor compartment were wrapped with Parafilm M^®^. After 6 h of application (application time was decided based on preliminary studies), formulation was removed; skin was cleansed with deionized water and placed back to the donor compartment. Samples from both the donor compartment (skin samples for dermatokinetic analysis) and receiving compartment (for estimation of permeation of CAR) were taken at pre-determined time intervals; 0.5, 1, 2, 4, 6, 8, and 24 h. In order to process the skin samples, these were washed with water three times for removal of excess formulation, followed by biopsy (biopsy punch; 10 mm diameter) (Stiefel, Middlesex, UK) to collect required skin sections. The skin was then added into glass tubes and heated at 60 °C in a water bath for 2–3 min. Epidermis was further separated manually from the dermal layers using tweezers. Thereafter, 500 µL methanol and stainless steel beads (5 mm diameter) were added in each layer followed by homogenization for 10 min at 50 Hz using Tissue Lyser LT (Qiagen, Ltd., Manchester, UK) to extract CAR from the skin. Homogenized samples were then centrifuged at 14800 rpm for 15 min and finally, a sample from supernatant was diluted with phosphate buffer (pH 7.4) before quantification of CAR using analytical method provided in Section 2.13. PK Solver software was used for data analysis by applying one-compartment open model and the kinetic parameters were calculated for both the epidermis and dermis. The samples taken from receiving compartment were analyzed for CAR permeation after making suitable dilutions with phosphate buffer (pH 7.4). Results for permeation were presented as drug permeated/unit area versus time. The following equation was used to calculate flux:

(5) $$J\mathrm{ss}=\left(\frac{dQ}{dt}\right)\mathrm{ss}\times 1/A$$

where *J*ss = steady state permeation flux (µg/cm^2^/h), *A* = Area of skin tissue, and (d*Q*/d*t*)ss = Amount of CAR permeating through skin per unit time at a steady state (µg/h).

#### 2.11.3. Skin Distribution Studies

To evaluate the distribution profile of CAR in different layers of the skin, only the final time point was selected i.e., 24 h. The skin samples were collected in the same way as described in Section 2.11.2. Following this, biopsied samples were frozen using liquid nitrogen then sliced into sections (50 µm each) using a Cryostat (Leica CM1900 Microsystems, Nussloch, Germany) till the whole skin was sliced. Four consecutive slices were gathered in one eppendorf tube. To extract CAR from the skin tissues, 500 µL of methanol was added, vortexed for 30 min, and then centrifuged at 14,800 rpm for 15 min. A sample of supernatant was then analyzed after making appropriate dilutions with phosphate buffer.

### 2.12. Determination of Antibacterial Effectiveness in an Ex-Vivo Pig Skin Wound Model

The ability of CAR and CAR-PCL NPs loaded hydrogel to reduce MRSA colonization was determined in an ex-vivo pig skin wound model using a previously reported method with slight modifications [6]. The skin samples were prepared following the same protocol mentioned in Section 2.11.1. The pig skin was cleansed with 70% ethanol and cut into equal circular patches of size 1.3 cm in diameter then was set into petri dishes having Kleenex paper (soaked in sterile water) in the bottom. Red hot brass knob was used to make burn wounds of size 5 mm in diameter (circular) and 0.5 mm deep. Morphology of the created skin wounds was observed using an optical coherence tomography (OCT) microscope (Michelson Diagnostics Ltd., Kent, UK). MRSA test strains were cultured and adjusted to a concentration of 2.0 × 10^5^ CFU/mL in a similar way as mentioned in Section 2.7.1. MRSA suspension (100 µL) was applied to all wounds. Petri dishes were covered with lids to establish a moist chamber, and then incubated at 37 °C for 4 h. CAR, blank PCL NPs, and CAR-PCL NPs loaded hydrogels were applied on infected wounds and the petri dishes were incubated for 18 h at 37 °C. After 18 h treatment, each skin wound was placed into 1.5 mL eppendorf tube followed by addition of 1 mL of sterile phosphate buffer (pH 7.4), and was homogenized utilizing Tissue Lyser LT (Qiagen, Ltd., Manchester, UK) at 50 Hz for 15 min to harvest bacteria from the skin. Finally, resultant bacterial suspensions were analyzed for surviving CFUs by inoculation on MHA plates followed by 48 h incubation at 37 °C. Skin wounds without application of hydrogels were run as the positive control and uninfected wounds were as the negative control. The experiment was performed in triplicate.

### 2.13. Instrumentation and Chromatographic Conditions of Analytical Method

The quantification of CAR was done using reversed-phase high performance liquid chromatography (HPLC) via Agilent Technologies 1220 infinity compacted LC Series (Agilent Technologies UK Ltd., Stockport, UK). The column used was Phenomenex LunaC18 ODS (1) column: 150 mm × 4.60 mm internal diameter, 5µm particle size (Phenomenex, Cheshire, UK). Acetonitrile: water (50:50 *v/v*) was used as mobile phase and was filtered using a 0.22 µm filter, and degassed by sonication (Decon â FS300b sonicating-water bath) prior to use for 1 h. 80 µL of injection volume and 1 mL/minute flow rate were used. A UV detector was used at 275 nm. A 50 µL rinse solution of acetonitrile was drawn into the injection needle between each sample run to ensure adequate cleaning. Validation of the analytical method was done in accordance with the International Committee of Harmonization (ICH) 2005.

### 2.14. Statistical Analysis

All tests/investigations were done in triplicate and data were manifested as a mean ± SD. Results were statistically analyzed by unpaired *t*-test, ANOVA, along with Bonferroni multiple comparison test (where appropriate) to determine statistical significance (specified at *p* < 0.05).

## 3. Results and Discussion

### 3.1. Statistical Analysis of Experimental Data by Design-Expert Software

Utilizing Central Composite Design (CCD) under Design-Expert^®^ (version 7, Stat-Ease, Inc. 2021 East Hennepin Ave., Suite 480 software) eleven formulations with varying concentrations of poly(ε-caprolactone) (PCL) (*X*_1_), surfactant (*X*_2_), and carvacrol (CAR) (*X*_3_) were successfully formulated and screened, as described in Section 2.2. The most desirable mathematical model was chosen on the basis of statistical goodness-of-fit which includes *F*-value, predicted and adjusted *R*-squared values, and adequate precision. Data was analyzed after confirmation of model significance in terms of ANOVA having *p*-value < 0.05. After careful consideration, the influence of each independent variable on various responses was determined.

#### 3.1.1. Effect of Independent Variables on Particle Size

Particle size is a key parameter in determining the physicochemical properties (e.g., drug loading, release profile and bioavailability) and physiological behavior (e.g., interaction with plasma components, phagocytosis, and uptake) of NPs [37]. Herein, the mean particle size of the eleven formulations was found to be in the range of 163.7 nm to 233.05 nm (Table 1), that is favorable for deeper skin penetration [38]. The quadratic model, having a value of *R*-squared (1.0000), is in equitable correlation with Adj *R*-squared (0.9997), and was found best fit for response *Y*_1_, in comparison to linear and two-factor models. To evaluate the significance of the quadratic model for quantitative effects of variables on response, ANOVA was carried out. The ANOVA indicated that the quadratic model was significant (*p* = 0.01) with the model *F*-value 3522.47. The low value 0.21% (desired less than 10%) of coefficient of variation (CV) evidently manifested high degree of precision along with considerable reliability of the experimental values. “Adeq Precision” measures the signal to noise ratio and value higher than 4 is preferable [39]. The obtained value, i.e., 176.298, indicated a satisfactory signal suggesting that predicted model might be useful to direct the design space. Based on data analysis, an empirical second order polynomial equation was obtained, which in the form of coded factors, is as follows:

Mean particle size (nm) *Y*_1_ = + 205.82 + 34.50*X*_1_ − 2.23*X*_2_ + 0.91*X*_3_ + 5.83*X*_1_*X*_2_ − 9.73*X*_1_*X*_3_ + 9.34*X*_2_*X*_3_ + 7.32*X*_1_^2^ − 10.9*X*_2_^2^ − 10.73*X*_3_^2^ (6)

Results of ANOVA suggested that particle size was significantly affected by PCL concentration (*X*_1_), interactive term of PCL and CAR concentration (*X*_1_*X*_3_), and polynomial models of Poloxamer^®^ 407 (*X*_2_^2^) and CAR concentration (*X*_3_^2^) with *p*-values < 0.05, while remaining term coefficients were not significant (*p*-values > 0.05).

As shown in Equation (6), the large positive coefficient for *X*_1_ demonstrated that increasing polymer content increased the particle size drastically (*p* = 0.0098). It can be ascribed to the point that increasing the polymer concentration increases the frequency of collisions between the particles during emulsification, which results in the fusion of semi formed particles, that subsequently increases particle size [40]. Moreover, higher polymer concentration improves the viscosity of the organic phase, that eventually reduces its diffusion rate towards the aqueous phase subsequently resulting in larger nanoparticles [41].

**Figure 2:**
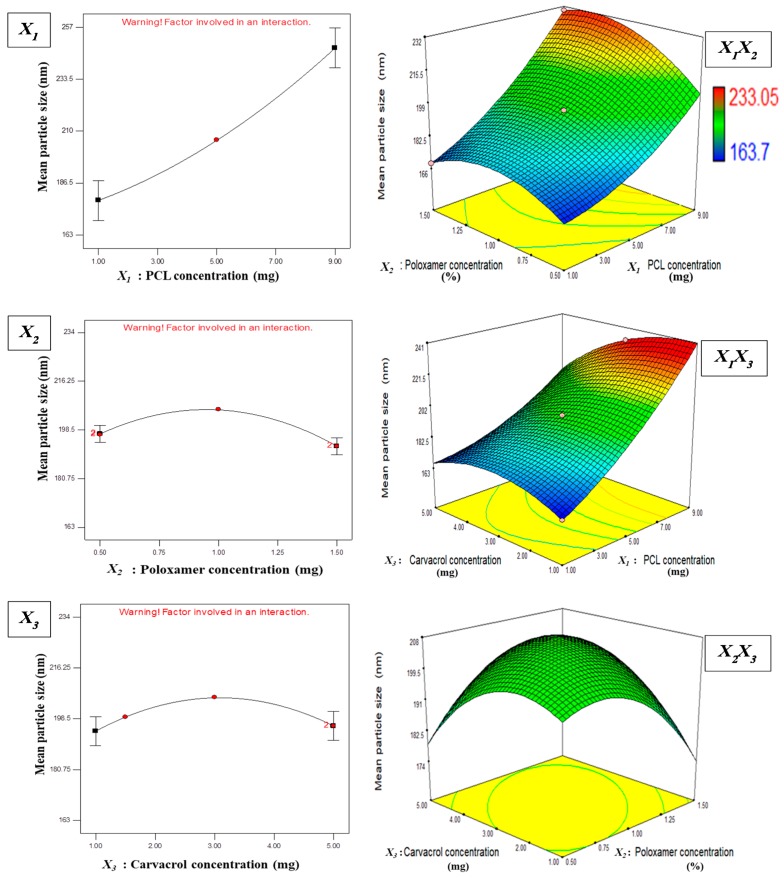
Response surface plots (2D and 3D) showing the influence of independent variables on particle size.

In the current study, Poloxamer^®^ 407 was used as a hydrophilic surfactant. Negative coefficient value for *X*_2_ with 0.07 *p*-value suggested that the Poloxamer^®^ 407 concentration has no impact on particle size. In Figure 2, it is evident that increasing the amount of Poloxamer^®^ 407 from 0.5% to 1%, had a positive effect on particle size, and later from 1% to 1.5% resulted in decreased particle size. In 3D surface plots, simultaneous effects of two variables can be observed. The curvature nature of plot *X*_2_*X*_3_ indicated strong interaction between the variables to influence particle size. Positive coefficient value for *X*_3_ indicated that concentration of carvacrol has direct relation with particle size, but that it is not significant (*p* = 0.25). Two and three-dimensional surface plots clearly explain the impact of various independent variables on particle size as shown in Figure 2. Intensity of red color represents increase in particle size with respective change in concentrations of PCL, surfactant, and CAR.

#### 3.1.2. Effect of Independent Variables on EE

The ability of NPs to encapsulate considerable amount of CAR is essential to produce required antibacterial activity. The EE% obtained for CAR was in the range of 27–89% and is presented in Table 1. In comparison to linear and two-factor models, a quadratic model having *R*-squared equal to 1.0000 and Adj *R*-squared of 0.9999 was found best fit for response *Y*_2_. The ANOVA indicated that the quadratic model is significant (*p* = 0.0077) and the model *F*-value is of 10232.62, which presented only a 0.77% chance of error. In addition to this, a very low value of CV, i.e., 0.33%, evidently suggested a high degree of precision and reliability of the experimental values. The value of Adeq precision, i.e., 324.539, indicated the usefulness of the predicted model for navigation of design space. Based on data analysis, the resultant equation obtained is given below:

Entrapment efficiency (%) *Y*_2_ = + 62.91 + 16.69*X*_1_ + 2.44*X*_2_ + 11.88*X*_3_ − 0.29*X*_1_*X*_2_ + 12.85*X*_1_*X*_3_ − 18.23*X*_2_*X*_3_ + 3.87*X*_1_^2^ − 1.32*X*_2_^2^ − 1.79*X*_3_^2^ (7)

The results of ANOVA suggested that EE% was significantly influenced by the linear coefficients (*X*_1_, *X*_2_, and *X*_3_) and the interactive coefficients (*X*_1_*X*_3_ and *X*_2_*X*_3_) with *p*-values < 0.05. As shown in Equation (7), the positive coefficient for *X*_1_ shows that the increasing concentration of hydrophobic polymer results in increased EE (*p* = 0.0098), that can be explained on the basis of the hydrophobic nature of CAR and its greater miscibility in the organic phase. Previous studies reported this trend for hydrophobic drugs, suggesting that the increased interactions with polymeric solution and increased consistency of organic phase reduce the partitioning of drug in aqueous phase, subsequently resulting in higher encapsulation in NPs [42].

The positive coefficient value for *X*_2_ suggested that Poloxamer^®^ 407 concentration has a significant effect on EE% (*p* = 0.03). This may be due to the addition of Poloxamer^®^ 407 into the aqueous phase which increases its viscosity, resulting in reduced diffusion of CAR into the aqueous phase, which in turn, increases EE.

**Figure 3:**
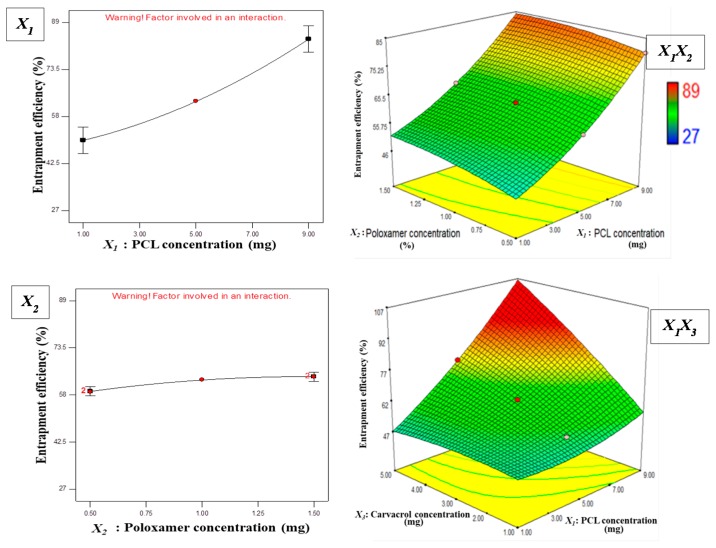

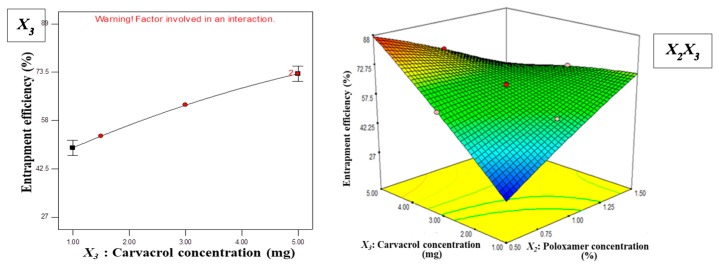
Response surface plots (2D and 3D) showing the influence of independent variables on entrapment efficiency.

The positive coefficient value for *X*_3_ indicated that concentration of CAR has direct relation with EE% (*p* = 0.009). As illustrated in Figure 3, by increasing initial content of carvacrol, the EE% increases. These results are in line with previously reported studies from other investigators, who reported that up to the optimum amount, with increases in initial drug content, EE also increases [43,44]. Two and three-dimensional surface plots distinctly illustrate the impact of various independent variables on EE% (Figure 3). The intensity of red color represents increase in the EE% with the respective change in concentrations of PCL, surfactant, and carvacrol.

### 3.2. Optimization and Validation

After analysis of polynomial equations presenting both independent and dependent variables, further optimization and validation was carried out. For this purpose, design expert software was utilized to get the best formula solution for CAR-PCL NPs development with desirable characteristics (minimal particle size and maximum EE). The composition of the optimized formulation was deduced as 5.40 mg/mL PCL, 0.5% Poloxamer^®^ 407, and 5 mg/mL CAR. The predicted values of *Y*_1_ and *Y*_2_ at these levels were 178.88 nm and 89% respectively, with desirability of 0.884 (having an 88.4% chance of producing the predicted results in terms of particle size and EE). Thereafter, CAR-PCL NPs were prepared according to the suggested optimal values and assessed for confirmation of predicted model. A very small variation between theoretical predictions and obtained results confirmed the validity of CCD utilized to get the desired CAR-PCL NPs formulation.

### 3.3. Physicochemical Characterization of Optimized CAR-PCL NPs

The particle size and PDI are essential parameters to determine width of size distribution and for subsequent drug permeation through skin. The optimized CAR-PCL NPs had a particle size of 190 nm which was in agreement with the predicted value (178.88 nm). Blank PCL NPs had slightly reduced particle size (184 nm), as compared to that of CAR-PCL NPs. A very low value for PDI (0.079 and 0.046 for blank and CAR loaded NPs, respectively) and unimodal shape of size distribution curves (Figure S1a,b), demonstrated the monodispersed and homogeneous nature of formulations. ZP is a key factor to evaluate the stability of colloidal dispersions [45]. The results of stability studies of CAR-PCL NPs loaded hydrogel demonstrated that the CAR-PCL NPs could maintain their properties over time (Table S4). It was observed that the ZP of CAR-PCL NPs (−17.7 mV) was increased, in comparison to blank NPs (−13.7 mV), indicating that CAR induced a more negative charge to the particles (Figure S2a,b). This is in line with previously reported studies [43]. Moreover, a negative surface charge is favorable for deeper skin penetration of NPs, as reported by previous studies [46].

**Figure 4:**
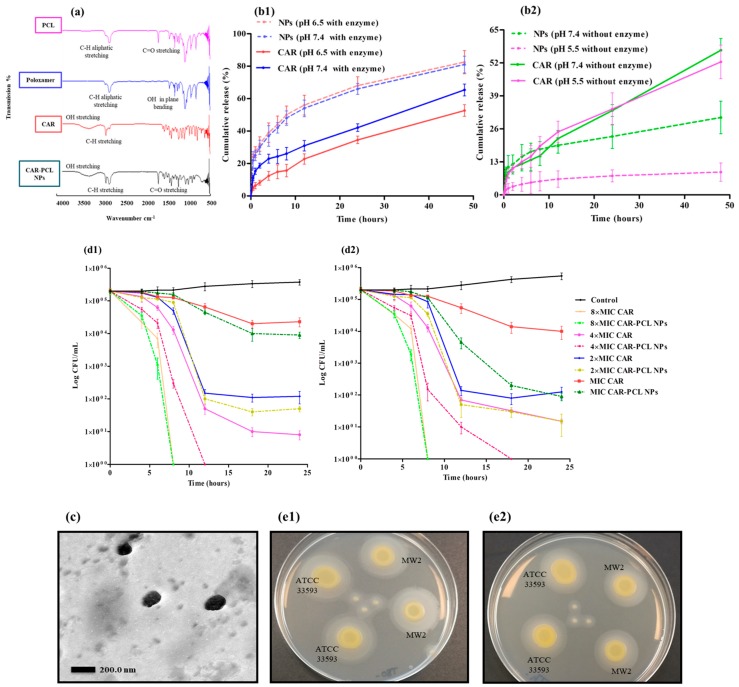
(**a**) FTIR spectra of CAR-PCL NPs and their components; (**b1**) in-vitro release profiles of CAR and CAR-PCL NPs in presence of bacterial enzyme (mean ± SD., *n* = 3); (**b2**) in-vitro release profiles of CAR and CAR-PCL NPs in absence of bacterial enzyme (mean ± SD., *n* = 3); (**c**) TEM image of CAR-PCL NPs; (**d1**) time kill assay of CAR and CAR-PCL NPs against ATCC^®^ 33593^TM^; (**d2**) time kill assay of CAR and CAR-PCL NPs against ATCC^®^ BAA-1707^TM^ (MW2); (**e**) Tween 80 agar based precipitation test; (**e1**) precipitation after 24 h of incubation: (**e2**) denser precipitation after 48 h of incubation.

The drug content entrapped in NPs is the most important parameter for their desired therapeutic activity. Optimized CAR-PCL NPs exhibited 83.28 ± 3.62% EE, which is comparable with the predicted value (89%). This EE is in correspondence with previous studies, which reported greater than 90% encapsulation of hydrophobic drugs in PCL NPs using this method of NPs formulation [47,48]. High percentage yield is desirable for a methodology to be economically beneficial. The percentage yield for CAR-PCL NPs was found to be 69.71 ± 4.19%. Representative TEM images of the optimized CAR-PCL NPs are shown in Figure 4c.

### 3.4. Fourier Transform-Infrared (FTIR) Spectroscopy

FTIR analysis was used to ensure that no chemical interactions had occurred between CAR and components used for NPs formulation. FTIR spectra of PCL, Poloxamer^®^, CAR, and CAR-PCL NPs are shown in Figure 4a. The spectra of PCL showed medium stretching around 2883.92 cm^−1^, owing to aliphatic C–H, and a strong stretching around 1725.59 cm^−1^ corresponding to the C=O group. The spectrum of Poloxamer^®^ showed medium stretching around 2971 cm^−1^ and 2882.46 cm^−1^ due to aliphatic CH groups, and in plane OH bending at around 1279.29 cm^−1^. These peaks are similar as reported by other studies [49]. CAR showed broad OH stretching at 3356.91, characteristic CH stretching at 2959.53 due to branched alkanes, CH deformation around 1459 to 1382 cm^−1^, C–C stretching of the aromatic ring at 1419.81 cm^−1^, and CO stretching at 1250.28 cm^−1^ [50]. All the representative peaks of CAR were the same in the NPs, confirming the presence of major functional groups, and the absence of any chemical interactions between CAR and the ingredients used for preparation of CAR-PCL NPs.

### 3.5. In-Vitro Release Kinetic Study of CAR-PCL NPs

Drug release studies provide information on the duration of drug availability. Release study was performed with CAR in pure form and CAR-PCL NPs at different pH conditions, in both the absence and presence of bacterial lipase enzyme (Figure 4b1,b2). The CAR has not shown any significant difference (*p* = 0.635) in release profiles at all the test conditions. In the presence of the enzyme, NPs showed a burst release of 27% and 25% in one hour and thereafter, with a slow release of up to 83.5% and 81% in 48 h obtained at pH 6.5 and 7.4, respectively. The burst release displayed by NPs can be ascribed to the low molecular weight of polymer and CAR and smaller size of particles. Polymer crystallinity associated with the low molecular weight of the polymer can also affect degradation kinetics, with more crystalline material presenting faster release than the amorphous one. It can be explained as microchannel structures forming in the crystalline matrix that may lead to a highly effective surface area for diffusion of the drug [51,52]. In contrast, CAR in the pure form has shown significantly less (*p* = 0.0263) release (52–65% in 48 h) than NPs; this less release could be ascribed to the hydrophobic nature of the CAR. There was no significant difference (*p* = 0.869) in total drug release from NPs at pH 6.5 and 7.4 in presence of enzyme. In comparison to this, NPs at the same pH 7.4 but in the absence of lipase, showed a significantly less release (*p* = 0.005) of 30.5% total in 48 h. This suggests that for polymer degradation kinetics, the presence of lipase is more influential than that of the pH of the medium. Importantly, at pH 5.5 in the absence of lipase, NPs showed negligible release (8.99%) in 48 h, as compared to pure CAR at same condition (*p* = 0.046) and NPs at pH 6.5 and 7.4 with lipase (*p* = 0.0001). These findings are also in line with those of Wu et al. [53] who reported that presence of the enzyme increases the degradation rate of PCL by 1000 fold, in comparison to degradation in the aqueous medium alone. A remarkable difference between the release of CAR in the presence and absence of lipase demonstrated the suitability of the designed system for potential selective delivery at infection sites. Hence, these results have proven the concept that loading of CAR into a responsive NPs system can avoid its release at non-specific sites. To anticipate the mechanism of release from formulations, various release kinetic models were applied using DD Solver software and the best-fit model was identified on the basis of highest *r*^2^ value. All formulations exhibited maximum linearity for Korsmeyer–Peppas model (Table S3). Furthermore, *n* values (diffusion coefficient) for all NPs formulations were less than 0.4, suggesting simple Fickian diffusion of CAR from polymer [54].

### 3.6. In-Vitro Antibacterial Assays

#### 3.6.1. Evaluation of Lipase Producing Activity

A precipitation test based on Tween^®^ 80 substrate was performed to detect the lipase production by MRSA test strains. Tween^®^ 80 was employed, as it comprises of esters of oleic acid, hydrolysis of which by bacterial lipases release fatty acids. Subsequently these fatty acids bind to the calcium available in the medium resulting in formation of insoluble white crystals around the inoculated colonies [55]. Both test strains showed white precipitation around colonies after 24 h of incubation (Figure 4e1) that was getting denser after 48 h (Figure 4e2) suggesting lypolytic activity of the bacterial enzyme. Based on these results, we can anticipate that bacteria at the site of infection will release lipase that can potentially degrade ester linkages in PCL NPs to release drug.

#### 3.6.2. Minimum Inhibitory Concentration and Minimum Bactericidal Concentration

The blank PCL NPs have not shown any antibacterial activity against MRSA strains. CAR-PCL NPs have shown an MIC value of 0.078 mg/mL against Staphylococcus aureus ATCC^®^ 33593^TM^, which is twofold, reduced in comparison to free CAR (0.156 mg/mL). Similarly, the MBC value of CAR-PCL NPs (0.156 mg/mL) was also twofold lower than MBC of free CAR (0.312 mg/mL). However, MBC values of both free CAR and CAR-PCL NPs were higher than their MIC values.

The CAR has shown same MIC value (0.156 mg/mL) and MBC value (0.312 mg/mL) against ATCC^®^ BAA-1707^TM^ (MW2) as for ATCC^®^ 33593^TM^. However, in case of CAR-PCL NPs the same MIC and MBC value (0.156 mg/mL) was found against ATCC^®^ BAA-1707^TM^ (MW2), showing >99.9% inhibition at its MIC value. Thus, obtained results indicated the bactericidal nature of both CAR and CAR-PCL NPs, as an antimicrobial is considered to be bactericidal if the ratio of MBC to MIC is ≤4 and bacteriostatic if it is >4 [56]. In contrast to free CAR, CAR-PCL NPs showed twofold reductions in MICs/MBCs of CAR when delivered via NPs. Thus, encapsulation of CAR into PCL NPs increased its anti MRSA potential. Juliane et al. obtained similar results, showing that microencapsulation of CAR in hydroxypropyl-beta-cyclodextrin significantly improved its antibacterial potential [57]. The enhanced antibacterial activity of NPs might be due to several reasons; however, as the primary site for antimicrobial action of CAR is at the membrane and inside cytoplasm of the pathogen, the NPs may have increased CAR access to these regions by increasing its solubility. Thus, these results highlight the potential of the developed CAR-PCL NPs to be used for enhanced antibacterial activity in addition to site-specific delivery.

#### 3.6.3. Killing Kinetics of CAR and CAR-PCL NPs

Time kill curves of CAR and CAR-PCL NPs against both MRSA strains are displayed in Figure 4d1,d2 (ATCC^®^ 33593^TM^ and MW2 respectively). As depicted, both CAR and CAR-PCL NPs have shown significant reduction in viable bacterial count for both ATCC^®^ 33593^TM^ (*p* = 0.0009) and MW2 (*p* = 0.0003) strains in comparison to the untreated control. In the case of ATCC^®^ 33593^TM^, CAR and CAR-PCL NPs have shown 90% and 95% reduction in viable count at their MIC (0.156 and 0.078 mg/mL respectively) in 18 h of incubation, respectively. CAR has shown >90% reduction in CFUs while CAR-PCL NPs have shown >99% inhibition in 12 h at 2 × MIC, in 8 h at 4 × MIC, and only in 6 h at 8 × MIC, indicating rapid killing at higher concentrations. In the case of ATCC^®^ BAA-1707^TM^ (MW2), although CAR and CAR-PCL NPs have the same MIC value (0.156 mg/mL), CAR has shown 72.5% inhibition in 12 h leading to 93% inhibition in 18 h, while CAR-PCL NPs have shown 97.7% reduction in the first 12 h leading to 99.9% in 18 h, suggesting he rapid killing rate of NPs. At higher concentration (8 × MIC), CAR-PCL NPs have drastically reduced the viable count to log 3 CFU/mL after 6 h, followed by complete bacterial inhibition in 8 h. At all MICs, CAR-PCL NPs have shown greater and rapid inhibition of bacterial growth in comparison to free CAR. This rapid killing is as important as the bactericidal nature of the compound, because the quicker the antimicrobial agent kills, the more efficiently it can block the biofilm formation.

### 3.7. Characterization of CAR-PCL NPs Loaded Hydrogel

The successful development of CAR-PCL NPs was accompanied by designing a suitable dosage form with suitable characteristics for skin application. For this purpose, Carbopol 934, owing to its non-irritant nature, eminent bio-adhesive properties, capability to formulate elegant pharmaceutical gels, and good stability upon storage was selected [24].

#### 3.7.1. Physical Appearance, pH, Drug Content and Spreadability of Hydrogel

The prepared hydrogels were found to be homogenous in consistency. Both the hydrogels were cloudy in appearance; however, CAR-PCL NPs loaded hydrogel was cloudier than CAR loaded hydrogel. The pH value for both the CAR and CAR-PCL NPs loaded hydrogels was found to be in close range of neutral pH (6.73 ± 0.17 and 6.54 ± 0.28 respectively), thus unlikely to induce pH induced skin irritation. The percentage of drug concentration in CAR-PCL NPs loaded hydrogel was calculated to be 88.45 ± 3.27% *w*/*w* depicting that a high content of CAR-PCL NPs was present in the final formulation. Measurement of spreadability is important to evaluate the ease of application of the formulation. Spreadability values were found to be 6.3 ± 0.82 cm/sec and 5.7 ± 0.03 cm/sec for CAR and CAR-PCL NPs loaded hydrogel respectively; which are in line with previously reported values indicating the maximum ‘slip’ and ‘drag’, and better spreadability [58].

#### 3.7.2. Rheological Evaluation

**Figure 5:**
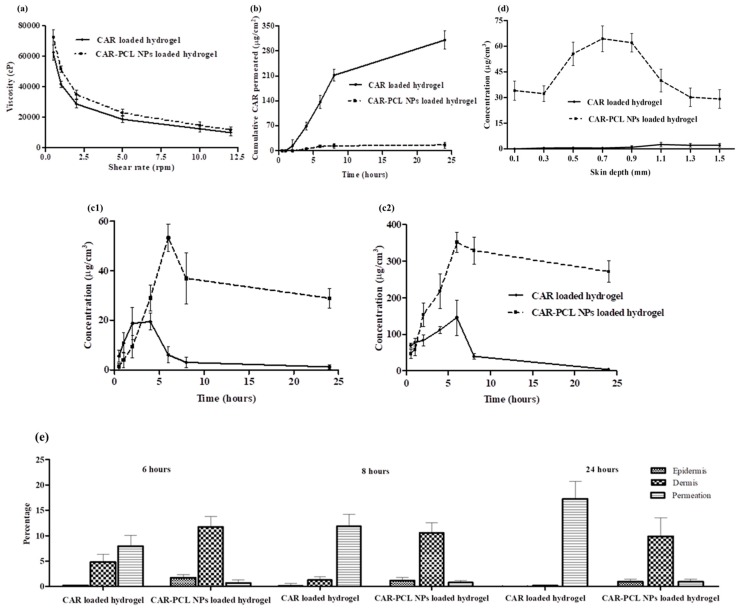
(**a**) Rheological properties of CAR and CAR-PCL NPs loaded hydrogel, (mean ± SD., *n* = 3); (**b**) cumulative CAR permeation through full-thickness pig skin followed by the application of CAR-PCL NPs loaded hydrogel in comparison with CAR loaded hydrogel (means ± S.D., *n* = 3); (**c**) CAR concentration and time profile in epidermis (**c1**) and dermis (**c2**) of excised neonatal pig skin; and (**d**) concentration of CAR in different layers of neonatal pig skin after 24 h of the application of CAR-PCL NPs loaded hydrogel in comparison with CAR loaded hydrogel (means ± S.D., *n* = 3); (**e**) comparison of CAR percentage permeated and CAR percentage retention in the epidermis and dermis layers of the skin treated with CAR and CAR-PCL NPs loaded hydrogel (means ± S.D., *n* = 3).

Estimation of the rheological property of the hydrogel is essential as it influences the ease of application, adhesion, and subsequent attachment to skin [59]. The flow curve was obtained by plotting shear speeds versus respective viscosities (Figure 5a). There was no significant difference (*p* > 0.05) found between the viscosities of CAR and CAR-PCL NPs loaded hydrogel. Evaluation of the rheogram indicated pseudo-plastic (non-Newtonian) behavior, desired for skin application because it can cover a maximum area.

#### 3.7.3. Extrudability and Bioadhesion Time of Hydrogel

CAR and CAR-PCL NPs loaded carbopol hydrogels showed excellent extrudability as the percentage of extruded gel was found to be 95.13 ± 0.24% and 94.48 ± 0.82% respectively. This is in agreement with previously reported studies suggesting the excellent extrudability of carbopol 934 based gels [60]. The retention time for CAR and CAR-PCL NPs loaded hydrogel was 54 ± 1.41 and 57 ± 0.51 min respectively. Acceptable adhesion time for NPs formulation was obtained due to appropriate viscosity of the formulation and adhesive properties of carbopol.

### 3.8. Storage Stability of Hydrogel

The stability of the hydrogel was investigated at three different conditions. No significant difference in parameters was observed at 4 °C and 25 °C. In contrast, at 40 °C variations in physicochemical parameters were prominent but this failed to reach statistical significance. Overall, the hydrogel did not exhibit any major changes that can induce instability. Thus, results of this study demonstrated the stability of formulations at all tested conditions, as presented in Table S4.

### 3.9. Ex-Vivo Dermatokinetic, Skin Deposition, and Distribution Studies

Dermatokinetic studies were conducted to determine the kinetic profile of CAR in epidermal and dermal layers. The results demonstrated that the concentration of CAR in the epidermis and dermal layers was considerably higher (*p* < 0.05) using CAR-PCL NPs loaded hydrogel as compared to CAR loaded hydrogel. Upon application of hydrogel on the skin, CAR penetrated into the skin more rapidly as compared to CAR-PCL NPs. However, free CAR permeated into the receiving chamber as rapidly as it penetrated into skin layers (a considerable amount was detected in the receiving chamber even after 2 h of application). Figure 5 describes the comparison of kinetic profile of CAR (concentration versus time) in both epidermis (Figure 5c1) and dermis (Figure 5c2) layers of porcine skin followed by the application of CAR-PCL NPs loaded hydrogel in comparison with CAR loaded hydrogel.

The dermatokinetic profile of CAR loaded hydrogel depicted that the *C*_max_ in the epidermis was found to be 17.40 ± 5.58 µg/cm^3^ after 2.07 ± 0.96 h. In the dermis, the highest concentration of 122.42 ± 18.81 µg/cm^3^ was achieved in 3.34 ± 0.42 h. The AUC0–24 of CAR in the epidermis was 122.15 ± 22.64 h. µg/cm^3^, and in the dermis was found to be 1109.76 ± 294.37 h. µg/cm^3^. With respect to CAR-PCL NPs profile in the epidermis, the *t*_max_ was found to be 11.40 ± 2.67 h with a concentration of 45.53 ±10.29 µg/cm^3^. While in the dermis, *C*_max_ (374.15 ± 31.86 µg/cm^3^) was achieved in 12.03 ± 1.90 h. The AUC0-24 in the epidermis was 862.31 ± 187.16 h. µg/cm^3^ and in the dermis was determined to be 7219.78 ± 493.08 h. µg/cm^3^. The values of *C*_max_ and AUC0–24 in the epidermis were found to be considerably lower (*p* < 0.05 each) than in the dermis. However, the values of all dermatokinetic parameters (Table S5) of CAR-PCL NPs were significantly higher (*p* < 0.05) in comparison with free CAR both in the epidermis and dermis, suggesting enhanced retention of CAR in skin layers after incorporation into NPs. The results of the permeation study further supported the retention potential of CAR-PCL NPs in skin layers. As demonstrated in Figure 5b, free CAR has shown significantly higher permeation (*p* < 0.0001) as compared to CAR-PCL NPs. A percentage comparison between CAR and CAR-PCL NPs loaded hydrogel in terms of CAR retention in skin layers and permeation at different time points (Figure 5e) indicated retention of CAR-PCL NPs in the dermal layers at all time points. At 6 h, following the application of CAR loaded hydrogel, only 0.2 ± 0.09% of total applied CAR was remained in the epidermis, 4.85 ± 1.52% in the dermis, and 7.93 ± 2.13% was permeated into the receiving chamber; while in the case of CAR-PCL NPs, 1.77 ± 0.56% was found in the epidermis, 11.71 ± 2.14% in the dermis, and very a negligible concentration was permeated 0.70 ± 0.13%. CAR-PCL NPs have shown retention in skin layers even after 24 h showing 0.96 ± 0.42% in the epidermis, 9.97 ± 3.70% in the dermis, and only 0.97 ± 0.34% permeation; while free CAR was almost completely permeated showing 0.04 ± 0.01% in the epidermis, 0.15 ± 0.06% in the dermis, and 17.73 ± 3.56% permeation. Considering the total amount of CAR delivered through hydrogels, 83.29 ± 3.15% of that was retained in dermis even after 24 h of application in case of CAR-PCL NPs, as compared to only 0.85 ± 0.14% in the case of free CAR.

With respect to the required concentration of CAR for antimicrobial activity, as described in Section 3.6.3, 0.156 mg/mL CAR-PCL NPs can kill >99.9% bacteria within 12–18 h. The *C*_max_ of CAR-PCL NPs achieved in the dermatokinetic study was 2.4 fold higher than required concentration and remained available at desired site for more than 24 h (mean residence time = 33.96 ± 11.30 h) demonstrating the suitability of CAR-PCL NPs loaded hydrogel for site specific delivery of CAR. In contrast to this, free CAR was required in higher concentrations (0.312 mg/mL) to show the same inhibitory effect, while the *C*_max_ of CAR obtained in dermatokinetic study was 2.6 fold lower than its required effective concentration. Moreover, its residence time was only 6.71 ± 0.86 h, that is not sufficient for CAR to show its desired antimicrobial activity. Hence, these results have proven the concept of this study that loading of CAR into NPs could be an effective approach to enhance its availability at site of action with improved antimicrobial activity. However, further in-vivo studies must be conducted to investigate the kinetic profiles of CAR in skin infection models.

The skin distribution of CAR and CAR-PCL NPs was then investigated in full-thickness porcine skin. Figure 5d demonstrates the concentration of CAR determined per cm^3^ in various depths of skin, after 24 h of application. Results of distribution study were in close agreement with dermatokinetic study, displaying improved distribution of CAR delivered through NPs. In case of free CAR distribution, as depicted in Figure 5d, CAR was detected in very minute concentrations only in deeper layers at 0.9 to 1.5 mm depths showing its clearance from skin layers after 24 h and confirming its permeation. In comparison to free CAR, CAR-PCL NPs showed entirely different distribution profiles. CAR in the form of CAR-PCL NPs was detected at all depths (0.1 to 1.5 mm) in considerable amounts, with higher concentration peaks showing at a depth of 0.5 to 1.1 mm. These results indicated that loading of CAR in NPs has significantly improved its sustained delivery into the range of dermal layers and hence, could potentially be used for variety of MRSA induced skin and soft tissue infections involving either only superficial skin layers (cellulitis, cutaneous abscesses) or deep dermal layers (erysipelas, infected wounds, burns).

### 3.10. Determination of Antibacterial Effectiveness in an Ex-Vivo Pig Skin Wound Model

**Figure 6:**
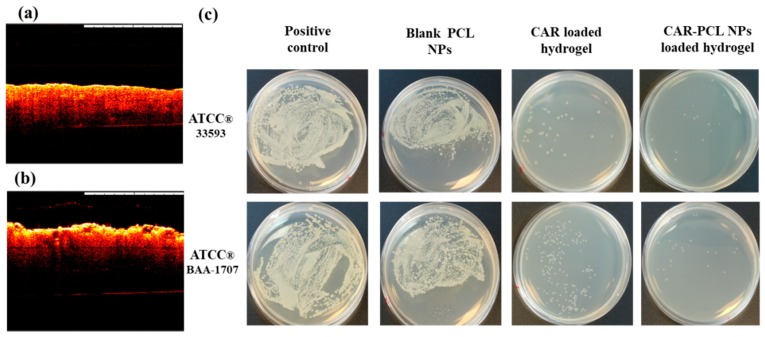
(**a**) Representative optical coherence tomography (OCT) image of normal neonatal porcine skin showing smooth surface of the skin, (**b**) representative OCT image of a burn wound showing uneven surface of the skin after burning. False colors were applied to the skin. The original OCT images can be seen in the supplementary information (Figure S3). The white scale bars at the top right represent a length of 1 mm. (**c**) Ex-vivo clearance of MRSA strains from infected pig skin wounds following the treatment with CAR, blank PCL NPs and CAR-PCL NPs loaded hydrogel.

Morphology of the burn wounds created in pig skin was observed using OCT and representative images are presented in Figure 6a,b. Post infection CFU counts in an ex-vivo pig skin wound model demonstrated a significant reduction in microbial burden in treated wounds in comparison to untreated ones (Figure 6c). Notwithstanding, there was no remarkable difference found between un-treaded wounds (positive control) and wounds treated with blank PCL NPs. The CAR loaded hydrogel showed 99.9 ± 0.09% reduction in microbial burden in wounds infected by ATCC^®^33593^TM^ and 99.62 ± 0.13% reduction in wounds infected by ATCC^®^ BAA-1707^TM^. Similarly, CAR-PCL NPs loaded hydrogel has shown 99.97 ± 0.05% reduction in microbial burden in wounds infected by ATCC^®^33593^TM^ and 99.95 ± 0.08% clearance in wounds infected by ATCC^®^ BAA-1707^TM^. This ex-vivo model has not presented any significant difference between the efficacy of CAR and CAR-PCL NPs loaded hydrogel in terms of antimicrobial activity because firstly, the amount of CAR applied in the form of CAR and CAR-PCL NPs were above their MBCs values, secondly, it was a closed system and all the drug applied was retained at the application site (that probably will not happen in an in-vivo open model). Although, current results demonstrated the bactericidal potential of CAR and CAR-PCL NPs loaded hydrogels in the ex-vivo model, however, it is required to investigate the antimicrobial effectiveness of developed formulations in an animal model to estimate actual behavior of formulations.

## 4. Conclusions

The current study demonstrated the successful development and characterization of an optimized bacterial enzyme responsive CAR-PCL NPs system utilizing a simple approach. In-vitro release studies in simulated infection media indicated the potential of the designed CAR-PCL NPs to release the drug in the presence of lipase producing bacteria. Moreover, antibacterial activity testing revealed that the antimicrobial activity of CAR was improved post loading into PCL NPs. Results of in-vitro/ex-vivo characterization of CAR-PCL NPs loaded hydrogel indicated its suitability in terms of dermal application and sustained drug delivery to desired site. The overriding advantage of this developed site-specific delivery approach presented here, in comparison to application of pure compound, lies in the NPs capacity to be retained in the skin for longer durations, which could potentially result in sustained desired antimicrobial effect avoiding multiple applications. Before this designed delivery platform can be used as a potential viable alternative treatment approach to that of conventional antibiotics for management of MRSA induced skin infections, however, further comprehensive studies are necessitated, including biocompatibility studies, and pharmacokinetics and pharmacodynamics studies in suitable animal infection models. Furthermore, based on the broad spectrum antimicrobial activity of CAR (reported in literature), efficacy of this system against other lipase producing bacteria involved in skin infections should also be investigated to fully explore its potential applications.

## Figures and Tables

**Table 1:** Composition, mean particle size and entrapment efficiency of CAR-PCL NPs, mean ± SD (*n* = 3).

Run	Independent Variables			Dependent Variables	
	*X*_1_ (mg/mL)	*X*_2_ (%)	*X*_3_ (mg/mL)	*Y*_1_ (nm ± SD)	*Y*_2_ (% ± SD)
F1	5.00	1.00	3.00	206 ± 2.61	63 ± 0.71
F2	5.00	1.50	3.00	192.5 ± 0.21	64 ± 2.13
F3	5.00	1.00	5.00	196 ± 3.60	73 ± 1.94
F4	9.00	1.50	1.00	229 ± 5.79	76 ± 3.21
F5	1.00	1.50	5.00	168.8 ± 1.76	30.5 ± 5.47
F6	5.00	1.00	1.50	199.1 ± 0.98	53 ± 2.91
F7	5.00	0.30	3.00	187.6 ± 0.56	57 ± 1.98
F8	9.00	0.50	3.00	233.05 ± 1.20	80 ± 3.16
F9	9.00	1.50	5.00	230.0 ± 0.21	89 ± 1.82
F10	1.00	0.50	1.00	163.7 ± 1.13	27 ± 4.37
F11	5.00	0.50	3.00	196.7 ± 2.89	59 ± 2.63

## Supplementary Materials

The following are available online at https://www.mdpi.com/1999-4923/11/11/606/s1; Figure S1: Particle size and PDI of optimized blank PCL NPs and CAR-PCL NPs, Figure S2: Zeta potential of optimized blank PCL NPs and CAR-PCL NPs, Figure S3: Original OCT images of normal porcine skin and burn wound. Table S1: Variables in CCD for optimization of CAR-PCL NPs, Table S2: The quantitative factor effects and associated *p* values for the responses, Table S3: Kinetic analysis of NPs at different pH conditions in presence and absence of enzyme representing correlation coefficient (*r*^2^) and *n* values using different kinetic equations, Table S4: Storage stability of CAR-PCL NPs loaded hydrogel for 3 months, mean ± SD (*n* = 3), Table S5: Dermatokinetic parameters of CAR and CAR-PCL NPs loaded hydrogel.
