# Supplementary material for: Enhancement in Site-Specific Delivery of Carvacrol against Methicillin Resistant Staphylococcus aureus Induced Skin Infections Using Enzyme Responsive Nanoparticles: A Proof of Concept Study
Source: Pharmaceutics. 2019 Nov 13;11(11):606. doi: 10.3390/pharmaceutics11110606 (PMC6921059; doi:10.3390/pharmaceutics11110606)
Supplement: Supplementary file 1 [file pharmaceutics-11-00606-s001.pdf]

pharmaceutics

MDPI

Enhancement in Site-Specific Delivery of Carvacrol against Methicillin Resistant *Staphylococcus aureus* Induced Skin Infections Using Enzyme Responsive Nanoparticles: A Proof of Concept Study: Supplementary materials

Maria Mir, Naveed Ahmed, Andi Dian Permana, Aoife Maria Rodgers, Ryan F. Donnelly and Asim.ur. Rehman

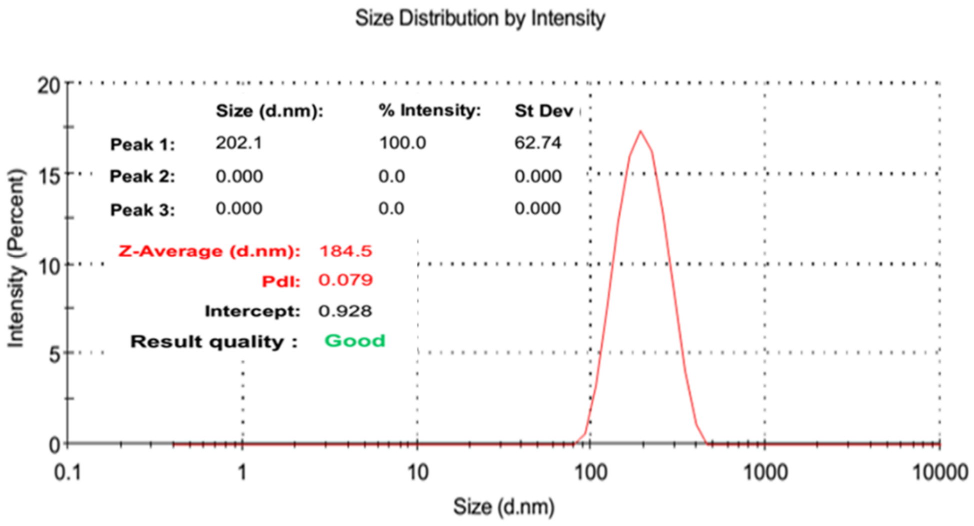

Figure S1(a). Particle size and PDI of optimized blank PCL NPs.

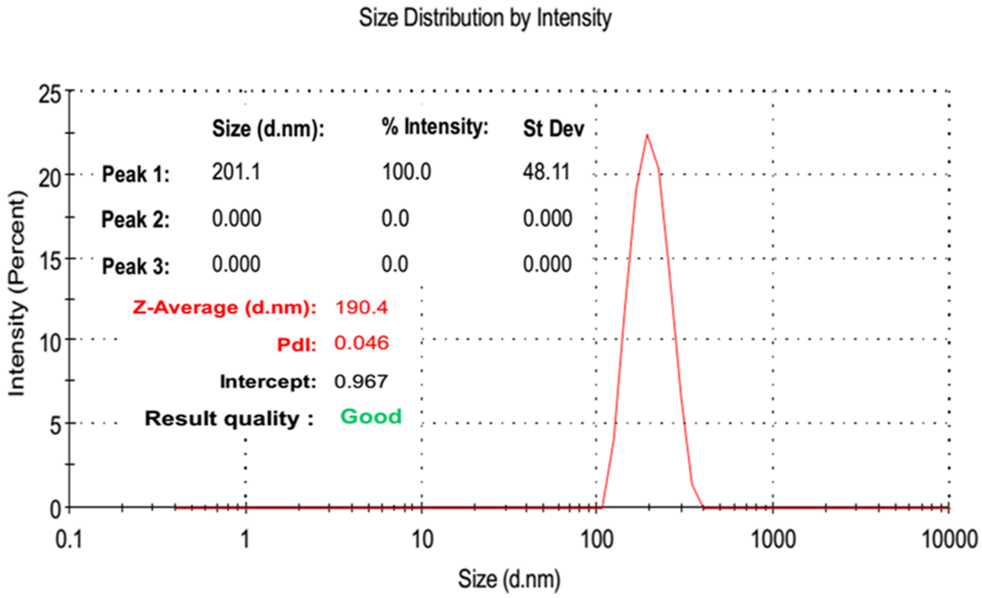

Figure S1(b). Particle size and PDI of optimized CAR-PCL NPs.

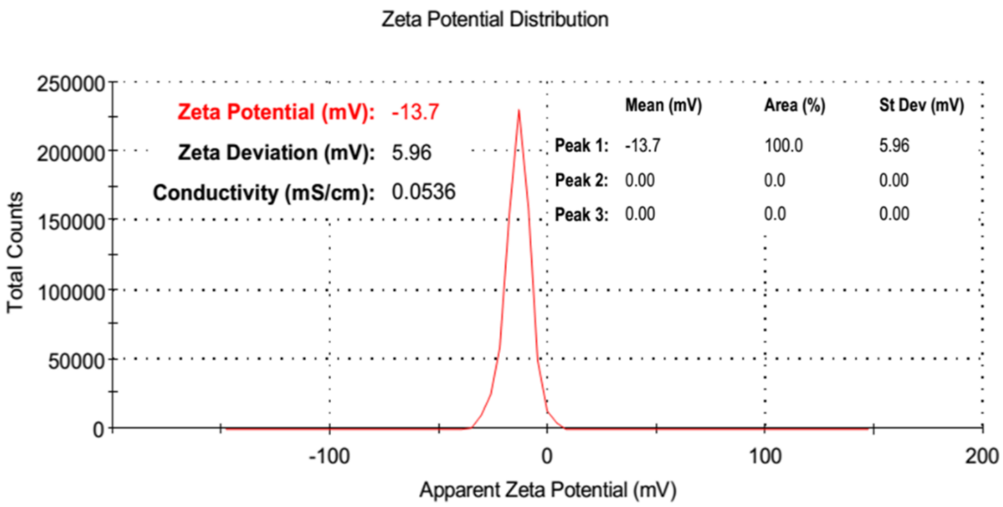

Figure S2(a). Zeta potential of optimized blank PCL NPs.

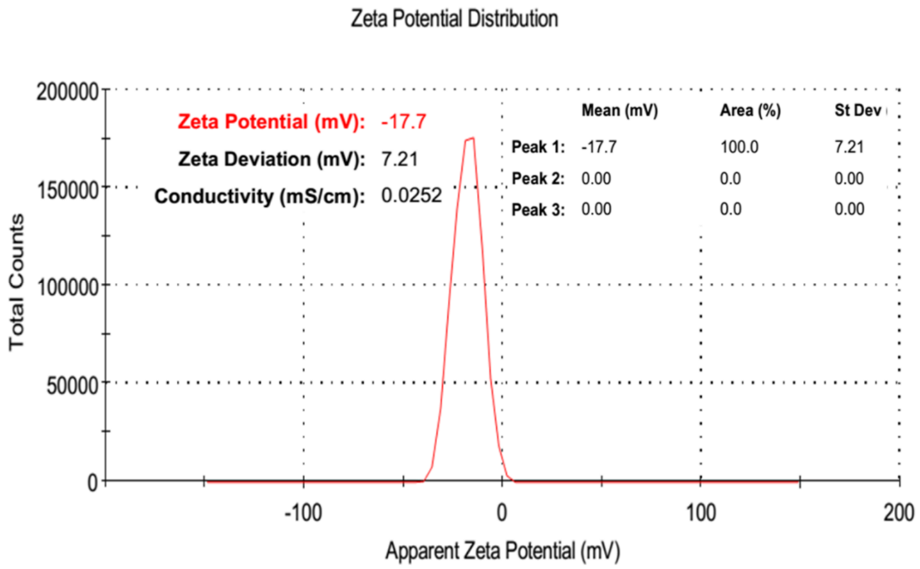

Figure S2(b). Zeta potential of optimized CAR- PCL NPs.

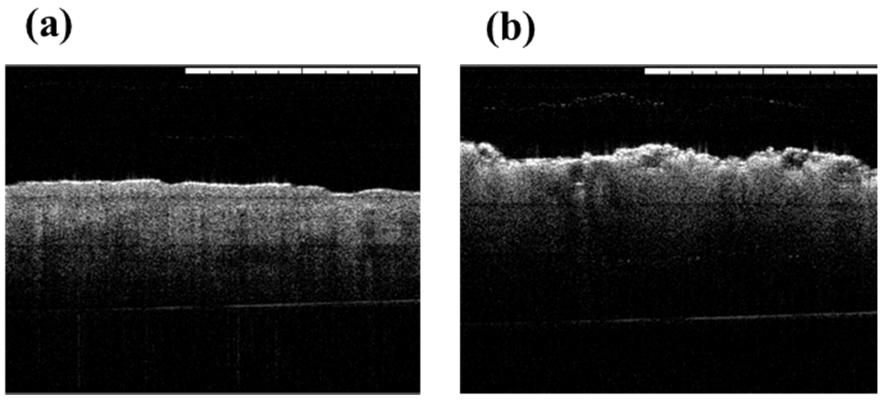

Figure S3 (a) The original OCT image of normal neonatal porcine skin showing smooth surface of the skin, **(b)** the original OCT image of burn wound showing uneven surface of the skin after burning. The white scale bars at top right represent a length of 1 mm.

Table S1. Variables in CCD for optimization of CAR-PCL NPs.

**Independent variables**	**Levels**		
	−1	+1	
X1: Polycaprolactone concentration (mg/mL)	1	9	
X2: Poloxamer® 407 concentration (%)	0.5	1.5	
X3: Carvacrol concentration (mg/mL)	1	5	
**Dependent variables**	**Constraints**		
	Low	High	Goal
Y1: Mean particle size (nm)	163.7	233.05	Minimize
Y2: Entrapment efficiency (%)	27	89	Maximize

Table S2. The quantitative factor effects and associated p values for the responses.

**Parameters**	***Y_1* (Mean Particle size)**		***Y_2* (Entrapement efficiency)**	
	**Effect**	**p-Value**	**Effect**	**p-Value**
X_1	34.5	0.0098*	16.69	0.0098*
X_2	−2.23	0.074	2.44	0.033*
X_3	0.91	0.2523	11.88	0.0099*
X_1X_2	5.83	0.0558	−0.29	0.4495
X_1X_3	−9.73	0.0497*	12.85	0.0183*
X_2X_3	9.34	0.0662	−18.23	0.0165*
X_1^2	7.32	0.0566	3.87	0.0521
X_2^2	−10.99	0.0203*	−1.32	0.0813
X_3^2	−10.73	0.0374*	−1.79	0.1082

*Significant value at $p < 0.05$.

Table S3. Kinetic analysis of NPs at different pH conditions in presence and absence of enzyme representing correlation coefficient (r^2) and n values using different kinetic equations.

Serial No.	Formulations	Zero order	First order	Higuchi	Hixson Crowell	Korsmeyer-Peppas	
		$Qt = K_0t$	$Qt = \ln Q_0 - K_1t$	$Mt / M\infty = K_Ht^{1/2}$	$Q_0^{1/3} - Qt^{1/3} = K_{HC}t$	$Mt / M\infty = Kt^n$	
		r^2	r^2	r^2	r^2	r^2	n
01	NPs (pH 6.5 with enzyme)	0.1785	0.6329	0.7913	0.5394	0.9961	0.293
02	NPs (pH 7.4 with enzyme)	0.1403	0.6289	0.8076	0.5311	0.9974	0.299
03	NPs (pH 7.4 without enzyme)	0.5068	0.2955	0.6529	-0.3662	0.9866	0.254
04	NPs (pH 5.5 without enzyme)	0.0143	0.0327	0.8512	0.0170	0.9967	0.318

Table S4. Storage stability of CAR-PCL NPs loaded hydrogel for 3 months, mean ± SD (n = 3).

Time in months	Synerisis/ Phase separation/ Grittiness			pH			CAR content (%)			Particle size (nm)			Extrudability (%)			Spreadability (cm/sec)		
Temp. ± 2°C	4	25	40	4	25	40	4	25	40	4	25	40	4	25	40	4	25	40
0	No	No	No	6.73 ± 0.17	6.73 ± 0.17	6.73 ± 0.17	88.45 ± 3.27	88.45 ± 3.27	88.45 ± 3.27	190.4 ± 2.35	190.4 ± 2.35	190.4 ± 2.35	94.48 ± 0.82	94.48 ± 0.82	94.48 ± 0.82	5.7 ± 0.03	5.7 ± 0.03	5.7 ± 0.03
1	No	No	No	6.73 ± 0.03	6.72 ± 0.12	6.71 ± 0.18	88.36 ± 2.13	88.25 ± 1.28	88.07 ± 4.27	193.2 ± 1.65	195.1 ± 0.74	197.2 ± 1.15	94.13 ± 0.62	92.42 ± 2.34	93.13 ± 1.22	5.8 ± 0.61	5.3 ± 0.25	6.1 ± 0.96
2	No	No	No	6.72 ± 0.06	6.72 ± 0.15	6.70 ± 0.01	87.85 ± 4.24	87.15 ± 4.17	86.84 ± 0.72	196.7 ± 0.81	203.6 ± 0.51	215.8 ± 0.84	93.05 ± 1.46	94.93 ± 1.65	92.21 ± 1.31	5.2 ± 1.89	6.2 ± 1.64	5.7 ± 1.05
3	No	No	No	6.72 ± 0.12	6.70 ± 0.08	6.69 ± 0.09	87.13 ± 5.06	86.92 ± 1.27	85.53 ± 2.39	201.3 ± 4.15	208.6 ± 2.43	223.3 ± 2.96	93.56 ± 0.98	93.62 ± 1.09	93.74 ± 1.17	5.9 ± 1.34	5.5 ± 0.95	6.3 ± 1.23

Table S5. Dermatokinetic parameters of CAR and CAR-PCL NPs loaded hydrogel.

Dermatokinetic parameters		t_{max} (h)	C_{max} (µg/ml)	AUC_{0-t} (µg/ml*h)	$t_{1/2}$ ka (h)	$t_{1/2}$ k10 (h)	MRT (h)
CAR loaded hydrogel	Epidermis	2.07 ± 0.96	17.40 ± 5.58	122.15 ± 22.64	1.85 ± 0.79	2.07 ± 0.96	5.66 ± 2.54
	Dermis	3.34 ± 0.42	122.42 ± 18.81	1109.76 ± 294.37	2.37 ± 0.25	2.62 ± 0.38	6.71 ± 0.86
CAR-PCL NPs loaded hydrogel	Epidermis	11.40 ± 2.67	45.53 ± 10.29	862.31 ± 187.16	7.17 ± 1.60	8.76 ± 2.25	22.19 ± 5.43
	Dermis	12.03 ± 1.90	374.15 ± 31.86	7219.78 ± 493.08	7.64 ± 2.97	8.86 ± 10.77	33.96 ± 11.30
